# SARS-CoV-2 testing strategies for a safe (post-)pandemic implementation of school music trips and their impact on participants’ health

**DOI:** 10.3389/fpubh.2025.1422243

**Published:** 2025-04-08

**Authors:** Linus Fritz Felix Möckel, Samipa Pudasaini, Kira Louisa Boldt, Fabian Holert, Anna Slagman, Stefanie Theuring, Frank Mockenhaupt, Martin Möckel

**Affiliations:** ^1^Department of Emergency and Acute Medicine, Charité – Universitätsmedizin Berlin, Corporate Member of Freie Universität and Humboldt-Universität zu Berlin, Berlin, Germany; ^2^Institute of International Health, Charité – Universitätsmedizin Berlin, Corporate Member of Freie Universität and Humboldt-Universität zu Berlin, Berlin, Germany

**Keywords:** school music trip, COVID-19 pandemic, SARS-CoV-2 PCR pool test, SARS-CoV-2 point of care test, hygiene concept, children’s health

## Abstract

**Introduction:**

During the coronavirus disease-19 (COVID-19) lockdowns, children repeatedly experienced social isolation. Dealing with the resulting post-pandemic health implications remains a challenge. The role of group recreational activities is crucial in promoting children’s health; however the implementation can encounter challenges, especially when infections such as COVID-19 are surging.

**Objectives:**

In this prospective observational study, we aimed to investigate whether safe cohorts can be created through appropriate test strategies to facilitate music trips during the COVID-19 waves. The primary outcome was the occurrence of positive cases during the journey. Secondarily, a survey was conducted to evaluate the physical and mental health status of participants before and after the first journey.

**Methods:**

Two school music trips were conducted. The first trip (T_1_) took place from 4 January 2022 to 9 January 2022, and the second trip (T_2_) from 3 January 2023 to 8 January 2023. For T_1_, central laboratory SARS-CoV-2 polymerase chain reaction (PCR) pool tests were performed before departure. For T_2_, prior point-of-care (PoC) PCR pool tests were conducted to validate the findings. A hygiene protocol was mandatory for T_1_ and recommended for T_2_.

**Results:**

Before T_1_, 95 volunteers underwent PCR laboratory pool testing, which revealed one positive COVID-19 case. During the travel, one student had a positive antigen test. Questionnaires for the mental health status were collected before T_1_ from 95 participants and again as a follow-up after T_1_ from 79 participants_._ There was a significant decrease in cold symptoms among students (*p* = 0.002). Following this, the perceived risk of infection significantly increased in the students’ group (*p* = 0.019). Additionally, anxiety symptoms [as measured using generalized anxiety disorder (GAD)-7 score] and the fear of getting infected marginally increased in students. All T_1_ participants indicated that they would be willing to attend a similar trip again. In the initial T_2_ pool testing, 88 participants took part. Two participants tested positive for SARS-CoV-2, with one solely showing signs of a subsiding infection and the other being highly infectious, which led to the exclusion of the highly infectious participant from the travel. During the trip and the follow-up period, no further cases were reported.

**Conclusion:**

Both testing concepts effectively identified positive “SARS-CoV-2 cases in advance and prevented transmissions, enabling safe school music trips during the winter. The use of PoC-PCR may be superior in terms of time efficiency and flexibility. Despite the increase in the perceived fear of infection among children, the overall experience of the journey was positive.

## 1 Introduction

As a result of the coronavirus disease-19 (COVID-19) pandemic, pursuing group leisure activities was temporarily restricted for children, and schooling lessons were moved to the digital space for the higher purpose of infection control (1). These contact restrictions and the subsequent social isolation caused an alarming increase in mental disorders in children and adolescents (2). After a partially effective containment of the COVID-19 pandemic across the world, society now faces the challenge of addressing the “silent pandemic” of mental health issues in youngsters (3–5). Only little research has been conducted on how to implement prevention and intervention strategies to counteract this rising trend of psychological diseases in the (post-)pandemic context (6, 7).

In addition to providing individual medical support, health policy associations consider group activities in schools and music to be particularly beneficial for the development of adolescents (8). In Germany and elsewhere, regular school trips are a fundamental part of the curriculum, often with the background of producing and performing music together in orchestras during the travel (6, 9). However, especially when wind instruments are in use, playing music in groups goes along with an elevated risk of aerosol transmission (10, 11). This risk is presumably further increased in the group travel setting of a school trip due to the close and regular all-day contact between the participants (12). Organizing school music trips while minimizing the transmission risk of SARS-CoV-2 is a challenging task, especially during the winter months. However, this type of children’s group activity bears the potential of positively influencing children’s (mental) health, as indicated in our previous study (6). Therefore, it should be further analyzed regarding various methods for its safe, season-independent implementation and its impact on participants’ health.

In a previous study by Pudasaini et al., we presented a successful hygiene and testing protocol to evaluate the feasibility of a school music trip by performing polymerase chain reaction (PCR) pool tests, which were analyzed in our central laboratory to rule out positive SARS-CoV-2 cases before the trip began (6). The study was performed during a COVID-19 wave when the Delta variant was still predominant (6, 13). However, the Omicron variant is known to have shorter incubation times (14). Thus, point-of-care (PoC) PCR strategies were considered and discussed as a possibly safer and well-established alternative to rapidly rule out highly sensitive COVID-positive cases (15). This testing strategy aimed to optimize the safety of the cohort safety by minimizing the time between the swab test and the start of the trip.

The objective of this study was to assess the safety and feasibility of two school music trips during the COVID-19 waves by comparing two main testing strategies: central laboratory PCR pool tests (trip T_1_) and PoC-PCR pool tests (trip T_2_). In addition to evaluating the testing strategies, the study aimed to capture the impact of this group music activity on the physical and psychological health of participants and to assess their overall experience during the trip.

## 2 Methods

### 2.1 Study period and population: T_1_ and T_2_

A prospective observational study was performed in one high school located in the southwest of Berlin (Steglitz-Zehlendorf) (16) with pupils coming from mainly two, overall five,surrounding schools. Two different periods were selected for T_1_ and T_2_. Both trips were planned to last 1 week taking place in 2022 (4th–9th January) and 2023 (3rd–8th January). On the day of the outward journey, the 7-day-incidence rate in Berlin was 286.8/100,000 for T_1_ (17) and 223.3/100,000 for T_2_ (18). The subsequent follow-up period lasted 5 days long. All children and music teachers who were permanent members of the main school’s internal three big bands received the offer to take part in the study. The participating teachers either worked at specific schools or were self-employed instrumental instructors. No further inclusion or exclusion criteria were applied.

### 2.2 Initial pool PCR tests: T_1_ and T_2_

**Figure 1:**
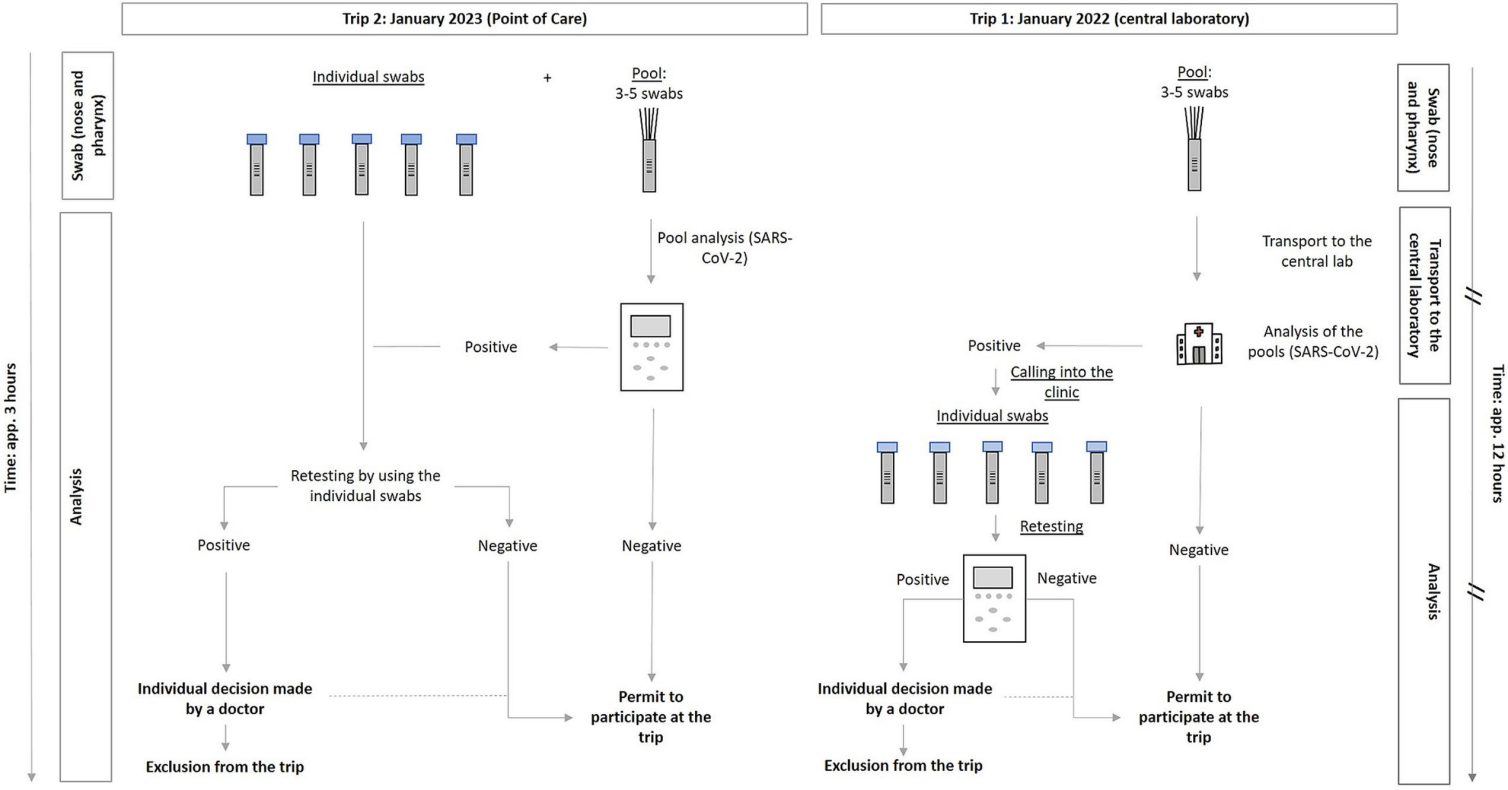
Diagram on the swab and analysis method performed via PoC PCR (left, T_2_: January 3–8, 2022), compared to the central laboratory PCR (right, T_1_: January 4–9, 2023). //: Marking of those steps that caused a temporal delay when analysis of PCR pool tests was performed in the central laboratory (T_1_). PCR, Polymerase Chain Reaction; T_1_, trip 1; T_2_, trip 2.

The participants in trip T_1_ functioned as an evaluation cohort, with central laboratory PCR pool tests for SARS-CoV-2 conducted before the journey as the reference standard. The concept of pool testing has been described elsewhere (19, 20). T_2_ was carried out for further validation and comparison. For this trip, a PoC-PCR pool testing method was implemented. For both journeys, the initial PCR tests were performed 1 day before the outward journey. The process of swab collection was done at the school site itself. In case of a positive sample, retesting all members of this specific pool was planned for the same day, therefore, definitely before the journey started. For participants of T_1_, retesting was done in our emergency department by performing individual cobas^®^ liat PCR tests (Roche Diagnostics, Mannheim, Germany) (21) to identify the SARS-CoV-2-positive cases. For trip T_2_, PoC-PCR devices were transported to one school, thus allowing the result receipt and retesting to happen directly on-site and in a shorter overall time span. For this, the cobas^®^ liat system (Roche Diagnostics) and, due to availability reasons, the combination assay cobas^®^ liat SARS-CoV-2 and influenza A/B (Roche Diagnostics) was used. During T_2_, individual swabs for a potential retest were already gathered simultaneously with the pool swabs but only processed in case one of the pool tests turned out to be SARS-CoV-2-positive. This aimed at further reducing any delay until receiving individual test results when in need of resolving a positive pool. Details regarding the testing procedures for T_1_ and T_2_ are comparatively presented in Figure 1.

### 2.3 Hygiene protocol and antigen tests: T_1_ and T_2_

To further reduce the SARS-CoV-2 transmission risk, the implementation of a hygiene concept was mandatory during the evaluation trip T_1_ and its follow-up period, also as part of the prevailing COVID-19 measures in Berlin schools at that time (see Supplementary File 1). Thus, students who participated in the T_1_ trip, but not the study, had to also follow the hygiene (and testing) protocol. The same rules were recommended for T_2_ but were not obligatory then, according to early 2023 COVID-19 school regulations. The hygiene protocol included hand hygiene, distancing, playing music in fixed groups, accommodation with no more than two students per room, the performance of daily rapid antigen tests (for trip and follow-up period; Novel Coronavirus 2019-nCoV tests by Hotgen, China) as well as the wearing of masks during the journey when in contact with people who were not part of the safe tested travel group itself. Any positive SARS-CoV-2 results were supposed to be reported to our senior medical supervisor, who was available remotely.

### 2.4 Questionnaires: T_1_

Prior to trip T_1_, all participants were able to fill out an online survey via our REDCap^®^ database. Each person received a pseudonym and was asked to use the same for the second questionnaire on the return journey. The aim was to match data in order to exploratively analyze developments in the course of the study. Surveys administered to students and teachers differed slightly.

Age and sex were the basic characteristics collected from the whole cohort in the first questionnaire. In the student population, we additionally determined the KIDSCREEN-10 score, a measuring instrument to assess the health-related quality of life in children (22). Furthermore, COVID-related information of all participants was gathered, including vaccination status, mask-wearing behavior (in the school/work environment), and prior traveling behavior to assess the overall COVID risk profile. In both questionnaires, at outward and inward journeys, we collected data on whether participants had contact with people who recently tested positive for SARS-CoV-2. Moreover, we extracted data on occurring physical symptoms before and after the trip, which included a catalog of 12 COVID-19-associated symptoms. An evaluation of the psychological state of our participants was done by applying the self-reported GAD-7 (generalized anxiety disorder) questionnaire for the assessment of anxiety symptoms (23). Also, all T_1_ participants were asked to estimate their subjectively perceived risk as well as their fear of getting a SARS-CoV-2 infection before and after the trip. For a general evaluation, we requested the whole cohort to inform us about their personal perception of this music trip (open question section) and whether they would decide to participate again under similar hygiene and testing conditions.

No questionnaires were administered to the participants of T_2_ since this school music trip solely served the purpose of validating the overall testing strategy for the creation of safe cohorts. However, all the students and teachers of the 2023 trip were encouraged to report respiratory symptoms or further reasons for suspected infections to our medical contact person.

### 2.5 Endpoints

The primary endpoint was the occurrence of positive SARS-CoV-2 cases during each music school trip and the subsequent 5-day follow-up periods. For trip T_1_, secondary outcomes were analyzed by administering questionnaires before and after participation. The sample size was limited to the number of participants in the trips, and therefore, no power calculation was feasible.

### 2.6 Statistical analysis: T_1_

**Figure 2:**
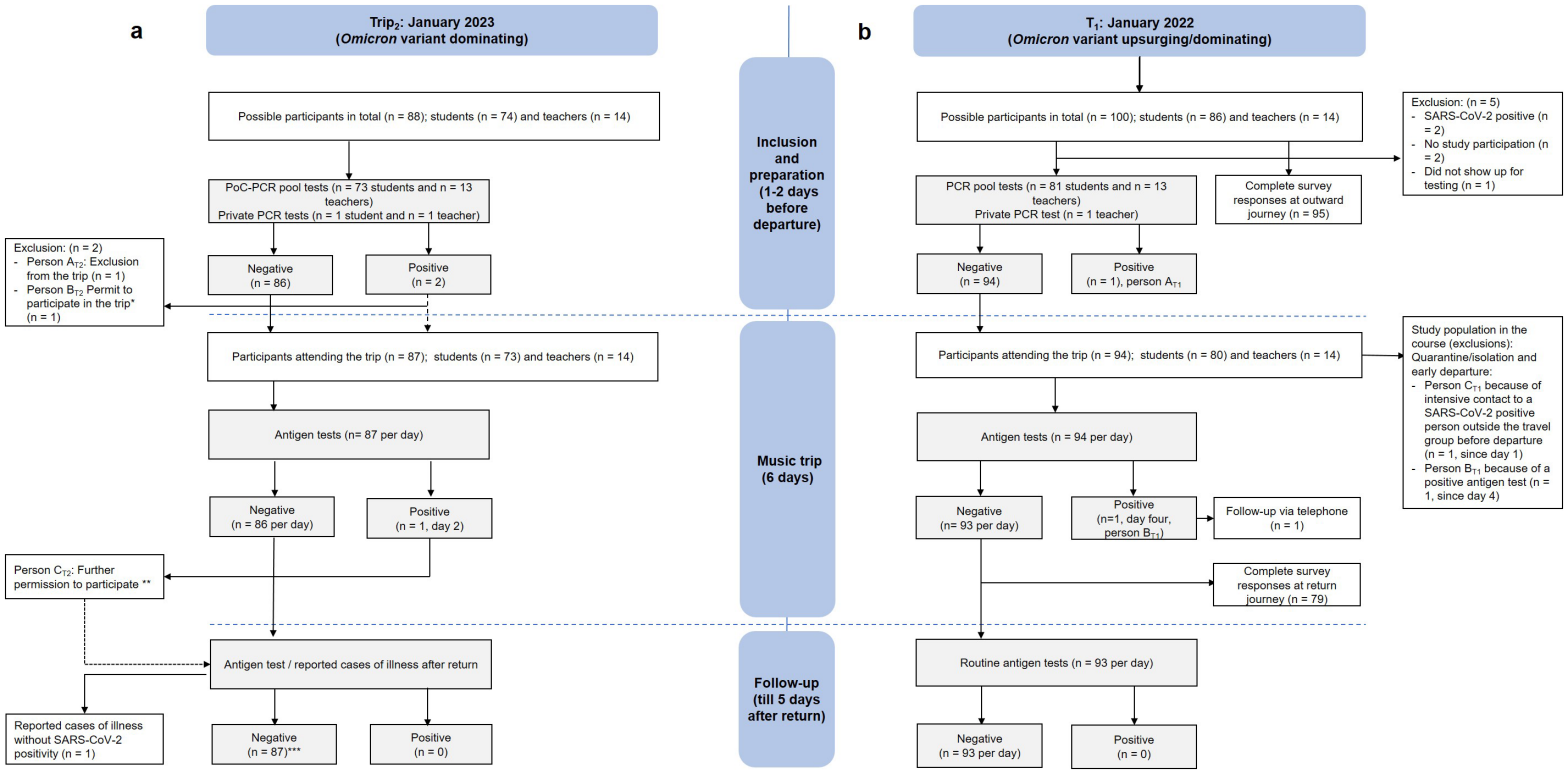
Diagram on the results of the testing procedure (initial tests, tests during the trip, and follow-up testing) of **(b)** T_1_ (right, January 4–9, 2022) and **(a)** T_2_ (left, January 3–8, 2023). * Participation despite a positive SARS-CoV-2 test, which (after a clinical evaluation) was found to be residually positive. ** After clinical evaluation (asymptomatic) and negative SARS-CoV-2 test results in a retest. *** Antigen test voluntarily. PCR, Polymerase Chain Reaction; PoC, Point of Care; T_1_, trip 1; T_2_, trip 2.

A descriptive analysis was applied for the evaluation of basic characteristics that were collected from surveys of the T_1_ cohort. Categorial variables of the descriptive analysis are reported as calculated proportions for categorical variables and mean (standard deviation, SD) for continuous variables. We extracted all survey responses that were filled in completely (*n* = 95 for departure and *n* = 79 for return; see Figure 2). An analysis of the KIDSCREEN-10 was performed by following the standard protocol of the *KIDSCREEN Group Europe* handbook (22). Threshold values for the categorization of the health-related quality of life (low/normal/high) are results of deviations from the mean value ±1/2 SD (*M* = 50, SD = 10).

When performing a comparative analysis of data from before and after the trip, we solely included questionnaires where a clear match of outward and inward survey responses was possible with the help of the distributed pseudonyms (*n* = 67). This was implemented for the following variables: all physical symptoms, anxiety symptoms (GAD-7 score), and individually perceived risk of the fear of getting infected with SARS-CoV-2. Here, a McNemar or Wilcoxon rank test was applied.

For both descriptive and statistical analysis, we made use of the IBM SPSS Statistics Version 26 (IBM Corp., Armonk, NY) software. A *p*-value of <0.05 was defined as statistically significant. The evaluation of open questions was done via qualitative content analysis.

### 2.7 Ethics approval and consent: T_1_ and T_2_

To perform this study, we received approval (EA2/091/20) from the ethics committee of Charité – Universitätsmedizin Berlin. It was conducted as part of the Berlin Corona School Study (BECOSS). Informed consent from all teachers and students (or their legal parent guardian) was needed for study participation but not necessary for attending the music trip itself.

## 3 Results

The trip T_1_ was performed between the January 3 and 8, 2022, with a follow-up period lasting until January 14, 2022. Out of 100 possible participants, 95 (81 students and 14 teachers) initially agreed to take part in the study and underwent the PCR pool testing procedure (*n* = 94) 1 day before departure or performed a private PCR test (*n* = 1). Two people declined to participate in both the study and the trip, while two were excluded as they were in isolation due to an ongoing SARS-CoV-2 infection. One student did not take the PCR test (see Figure 2).

For trip T_2_, 86 people out of possibly 88 participated in the initial pool testing, while two performed private PCR tests. Ultimately, 87 of them went on the music trip itself (73 students and 14 teachers), which took place between the January 4 and 9, 2023, similarly with a subsequent 5-day follow-up period (see Figure 2).

### 3.1 PCR pool test and antigen test results: T_1_ and T_2_

In our T_1_ cohort, we had two positive SARS-CoV-2 cases. The first participant (A_T1_) was detected via a positive pool PCR test, which was subsequently confirmed by a positive individual PCR test. That student was excluded from the trip and sent into isolation. The second positive test was discovered on day four of the music trip with a SARS-CoV-2 antigen test as part of the obligatory daily testing protocol. To confirm this result, an additional PCR test was performed. This student (B_T1_) was isolated in a separate hostel room and picked up early. A follow-up telephone call was later performed with this student B_T1,_ which revealed, as a possible source of infection, a contact with an external person who was not part of our journey group 2 days before the beginning of our study period. A virus variant analysis pointed to Omicron in both cases (A_T1_ and B_T1_). Until the end of our observation period, no one else in the travel group was infected, neither because of the two positive cases in our study group nor after having had contact with positive-tested (external) people before and/or after the trip. Thirdly, after a careful evaluation by our medical supervisor, another student (C_T1_) was isolated and sent home early as this participant was found to be having had an intensive SARS-CoV-2 contact in the days immediately before the journey (see Figure 2). However, he did not turn COVID-positive until the end of our observation span.

For T_2_, two positive PoC- PCR pool tests for SARS-CoV-2 (and influenza A) were registered 1 day before the start of the trip. Within the period of a few hours, we subsequently retested all individual swabs of the respective positive pools and detected two participants with a positive SARS-CoV-2 viral load (participants A_T2_ and B_T2_) as well as a further participant with a positive influenza A result. Due to a Ct (cycle threshold) value of 17, participant A_T2_ was considered highly infectious and was, thus, sent to home isolation. Participant B_T2_ (Ct = 33) was tested repeatedly, also on the following day, and received a clinical assessment. Based on a high Ct value of 33 and 31.9 and the health condition itself, this participant was classified as residually positive but not contagious by our medical supervisor and was allowed to join the travel group. The rapid antigen tests that were performed during the trip itself showed a single positive SARS-CoV-2 result (person C_T2_), which was interpreted as a false-positive due to multiple following retests being clearly negative and based on the simultaneous absence of symptoms. Altogether, after initially ruling out the highly positive participant A_T1_, no further SARS-CoV-2-positive findings were detected or reported during the journey or in our 5-day follow-up period (see Figure 2).

### 3.2 Evaluation of questionnaires: T_1_

All 95 participants from T_1_ who attended the initial PCR pool test also filled in the first questionnaire. After the trip, we received 79 follow-up survey responses (see Figure 2).

The basic characteristics of the T_1_ study population are provided in Table 1.

**Table 1:** Basic characteristics, the KIDSCREEN-10 score for children, information on school/work environment, and SARS-CoV-2-related data of T1 participants.

Basic characteristics		
Variables	Students	Teachers
Survey responses	**81**	**14**
Age in mean value (absolute number), standard deviation	**14.1** (79/81), 1.9	≤ 30: **50.0** (7/14)
		31–40: **21.4** (3/14)
		41–50: **7.1** (1/14)
		51–60: **13.3** (2/14)
		≥ 60: **7.1** (1/14)
Gender in % (absolute number)		
Women	**41.3** (33/80)	**21.4** (3/14)
Men	**57.5** (46/80)	**78.6** (11/14)
Diverse	**1.3** (1/80)	0

KIDSCREEN-10		
Health-related quality of life in % (absolute number)		
Low	**28.4** (23/81)	n. a.
Normal	**32.1** (26/81)	n. a.
High	**39.5** (32/81)	n. a.

School/work environment		
Mask wearing* in % (absolute number)		
In class	**98.9** (80/81)	n.a.
In the school’s hallway	**93.8** (76/81)	n.a.
During work	n.a.	**85.7** (12/14)
During breaks/in the school yard	**46.9** (38/81)	**71.4** (10/14)
On the way to school/work	**49.4** (40/81)	**57.1** (8/14)

SARS-CoV-2 based data		
Vaccination status in % (absolute number)		
Second vaccination ✓	**67.9** (55/81)	n.s.
Third vaccination ✓	**12.3** (10/81)	**85.7** (12/14)
Travel behavior in % (absolute number)		
Return from abroad - last 4 weeks	**8.6** (7/81)	**7.1** (1/14)
Contacts to SARS-CoV-2 positive cases in % (absolute number)		
On outward journey	**9.9** (8/81)**	0
On outward journey	**6.0** (4/67)***	**11.1** (1/9)***
On return journey	**31.3** (21/67)****	**50.0** (5/10)****

If not specified otherwise, percentage frequencies (bold) and absolute values are reported. *: Multiple answers were possible; **: outside of school during the 14 days before the study period; ***: outside of the travel group during the 14 days before return journey; ****: inside the travel group during the 5 days before return journey; variations in survey answers occur due to incomplete data provided by participants. n.a., not asked for; n.s., not shown (as value was implausible).

Data about the age of participating teachers was collected in four categories. Additionally, an assessment of our cohort’s SARS-CoV-2 risk profile for a possible infection, including vaccination status, was conducted. In the student group, 5% (4/80) tested positive in a PCR test and 1.3% (1/81) in a positive antigen test in the past. Also, as part of evaluating the SARS-CoV-2 risk profile, we requested the participants to report whether they returned from a foreign country in the 4 weeks before the start of the study or had contact with a COVID-positive person within the 14 days before the study period (outside of the school setting).

We compared reported symptoms in our T_1_ study population from data before and after the school music trip. As demonstrated in Table 2, signs of cough, headache, exhaustion, limb pain, and sore throat slightly increased during the trip. However, the category “symptoms of a cold” was solely reported significantly less often after students attended the journey (*p* = 0.002, McNemar test).

**Table 2:** Physical symptoms of students and teachers before and after the T1 trip.

Physical symptoms				
Variables*	Students		Teachers	
	Outward	Return	Outward	Return
Fever	**0**	**0**	**0**	**0**
Cough	**7.1** (4/56)	**10.3** (6/58)	**11.1** (1/9)	**11.1** (1/9)
Dyspnoea	**6.9** (4/58)	**5.2** (3/58)	**0**	**0**
Exhaustion	**22.8** (13/57)	**26.8** (15/56)	**22.2** (2/9)	**0**
Headache	**22.8** (13/57)	**33.9** (19/56)	**22.2** (2/9)	**11.1** (1/9)
Loss of taste and smell	**0**	**0**	**0**	**0**
Chills	**1.8** (1/57)	**1.8** (1/57)	**11.1** (1/9)	**0**
Symptoms of a cold	**31.6** (18/57)	**10.5** (6/57)**	**22.2** (2/9)	**11.1** (1/9)
Pressure on the chest	**3.5** (2/57)	**0**	**0**	**0**
Limb pain	**7.3** (4/55)	**8.8** (5/57)	**11.1** (1/9)	**11.1** (1/9)
Sore throat	**3.6** (2/56)	**10.3** (6/58)	**11.1** (1/9)	**11.1** (1/9)
Diarrhoea	**1.7** (1/58)	**1.7** (1/58)	**11.1** (1/9)	**11.1** (1/9)

Percentage frequencies (bold) and absolute values are reported. *: Multiple answers were possible; variations in survey answers occur due to incomplete data provided by participants. **: Significant decrease over the trip (*p* = 0.002, McNemar test); all other values show no significant increase/decrease.

Our GAD-7 score analysis revealed a slight overall increase in anxiety symptoms when comparing the status before with that after participation in the journey (*M* = 0.29, SD = 0.53). The reporting of anxiety increased for 10 students (18.1%, 10/55) while the score decreased for six (10.9%, 6/55) and stayed constant for 39 (70.9%, 39/55, see Table 3). In the group of teachers, a decrease in anxiety symptoms was visible, with three reporting less fear (33.3%, 3/9), six (66.6%, 6/9) keeping the same GAD-7 score, and no one presenting with an increased level of anxiety after the music trip. A distribution of absolute numbers for each category, from non-existent to severe anxiety symptoms, is shown in Table 3. The individually perceived risk and fear of getting infected with SARS-CoV-2 is also displayed in the table. Slight but non-significant increasing rates regarding the fear of SARS-CoV-2 infection were registered in the group of children. In terms of the subjectively perceived risk of infection, the detected rise during the trip was measured as statistically significant (*p* = 0.019, Wilcoxon rank test). A summed-up number of 18 students (31.0%, 18/58) reported a perceived higher infection risk in our follow-up survey than before attending the trip.

**Table 3:** Mental health of students and teachers before and after the trip T_1_.

**Psychological symptoms**				
	**Journey**			
Variables*	**Outward**	**Return**	**Outward**	**Return**
GAD-7 score in % (absolute number) Anxiety symptoms	Students (*n* = 55)		Teachers (*n* = 9)	
No to minimal (0–4 points)	**81.8** (45)	**74.5** (41)	**66.7** (6)	**88.9** (8)
Mild (5–9 points)	**14.5** (8)	**21.8** (12)	**11.1** (1)	**11.1** (1)
Moderate (10–14 points)	**0**	**3.6** (2)	**0**	**0**
Severe (15–21 points)	**3.6** (2)	**0**	**22.2** (2)	**0**

Perceived *fear* of getting infected in % (absolute number)	Students (*n* = 58)		Teachers (*n* = 9)	
None	**31.9** (9)	**18.9** (11)	**0**	**11.1** (1)
A bit	**50.0** (29)	**36.2** (21)	**55.5** (5)	**44.4** (4)
Modest	**29.3** (17)	**43.1** (25)	**22.2** (2)	**22.2** (2)
Strong	**5.**1 (3)	**0**	**22.2** (2)	**22.2** (2)
Very strong	**0**	**1.7** (1)	**0**	**0**

Perceived *risk* of getting infected in % (absolute number)	Students (n = 58)		Teachers (n = 9)	
Very low [1]	**15.5** (9)	**12.0** (7)	**0**	**33.3** (3)
Rather low [2]	**44.8** (26)	**36.2** (21)	**55.5** (5)	**11.1** (1)
Modest [3]	**34.4** (20)	**39.6** (23)	**22.2** (2)	**44.4** (4)
Rather high [4]	**5.1** (3)	**12.0** (7)	**22.2** (2)	**22.2** (2)
Very high [5]	**0**	**0**	**0**	**0**
Mean perceived risk	**[2,29]**	[2,51]**	**2,66**	**2,77**

Percentage frequencies (bold) and absolute values are reported. Anxiety symptoms were analyzed by using the GAD-7 score. Data on the following sub-categories were collected: (a) nervousness, (b) inability to stop worrying, (c) worrying too much, (d) having trouble relaxing, (e) restlessness, (f) being easily annoyed/irritable, and (g) fear as if something awful could happen (20). Also, the subjectively perceived risk of SARS-CoV-2 infection and fear of infection were evaluated. Numbers are assigned to the possible answers in square brackets, and the mean value of the given answers was calculated. *Variations in survey answers occur due to incomplete data provided by participants. **Significant increase over the trip (*p* = 0.019, Wilcoxon rank test); all other values show no significant increase/decrease. GAD-7 score, generalized anxiety disorder score.

**Figure 3:**
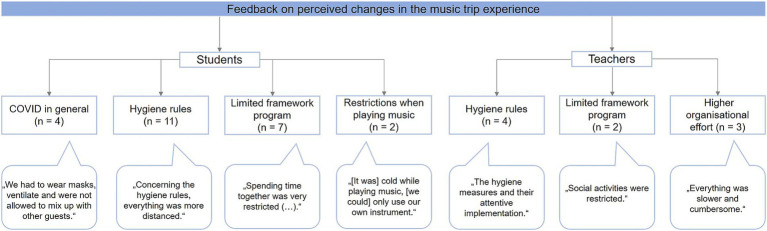
Answers from the open-question section concerning changes in the T_1_ journey experience compared to (pre-pandemic) music trips.

After returning from the trip, 59.7% (37/62) of the students and 36.4% (4/11) of the teachers described no relevant differences in the music trip experience compared to prior pre-pandemic journeys. Of the changes noted by the study population, constant hygiene measures (teachers: *n* = 4, students: *n* = 11) and restrictions concerning the framework program (students: *n* = 7) were mentioned most frequently. Details about the analysis of this open-question section are provided in Figure 3. Overall, the whole study population (teachers: 11/11; children: 68/68) reported that they would want to participate in such a musical group activity under similar conditions again.

## 4 Discussion

This observational study evaluated the safety and feasibility of 1-week school music trips during COVID-19 waves in January 2022 and 2023 by comparing the method of initial central laboratory PCR pool testing and PoC-PCR pool testing strategy to create safe cohorts. Our findings confirm the safety regarding the detection and avoidance of further COVID-19 cases during the trips. However, the PoC-PCR method may be considered superior in terms of feasibility due to its flexible usage and ability to provide rapid results. The overall perception of the T_1_ trip experience was, without exception, positive, although the effects on health varied between children and teachers.

### 4.1 PCR testing strategies for safe cohorts

This study confirms the findings of Pudasaini *et al.*, who conducted central laboratory PCR pool tests in a summer cohort with a low prevalence of SARS-CoV-2 (6). In our T_1_ group, the initial testing procedure allowed an early detection and exclusion of the SARS-CoV-2 positive person A_T1_, which, in turn, prevented any further transmissions in the traveling group. In comparison, the newly implemented PoC-PCR pool testing strategy, which was used for trip T_2_, similarly proved to be reliable and safe in detecting positive SARS-CoV-2 cases in advance and, on top of that, addressed two major obstacles:

This includes, firstly, the short incubation period of the then dominantly present Omicron variant of SARS-CoV-2 (14). With our pooled PoC measurements, which mostly have a turnaround time of 20 min (24), testing was possible on short notice, right before the start of the school trip, but still provided very reliable outcomes due to its high testing sensitivity (15). Longer swab-to-trip times could have increased the risk of newly occurring, uncaptured infections in the time after testing and before the trip started. In addition to this high time efficiency, the on-site offline PoC-PCR measuring technique was independent of (hospital-internal) laboratory infrastructure, enabling the test personnel to travel flexibly to an external school location.

Secondly, a major obstacle in the implementation of both cohorts was the fact that they were carried out in the winter months, a time that is characterized by a surge in COVID-19 cases (25). As demonstrated during trip T_2_, simultaneous co-testing for influenza A/B and the respiratory syncytial virus, via the use of combination assays, could provide even greater protection for future school cohorts since they may help reduce the transmission risk of a broad spectrum of seasonally fluctuating respiratory tract pathogens and not solely of SARS-CoV-2 strains.

### 4.2 PCR testing strategies for safe cohorts: feasibility

Due to the high acquisition costs of a PoC-PCR device (26) and the need for trained specialist staff to operate it, establishing a central testing service site appears to be the more pragmatic and sustainable option for future social activity implementations. For instance, it would be conceivable that the state’s public health service may be responsible to provide mobile testing personnel and devices to all inner-city school cohorts that show interest in increasing the safety of their group leisure activities during the times of high prevalence of COVID-19 as well as for respiratory viruses such as influenza A/B. This applies especially to school music trips or sports events, which come along with a comparatively high risk of infection transmission, especially during the cold season (10, 27, 28). The public health services in Germany explicitly define the protection of health in children and adolescents as one of their main objectives (29); therefore, promoting PoC-PCR testing options for schools could fall under this objective. Considering the costs and efforts required to implement testing services, it is likely that not all school trips and events could be supported. Focusing on events with a high potential for infection such as music and sports, as well as events including vulnerable cohorts such as physically impaired children, would likely be the most effective approach.

### 4.3 SARS-CoV-2 risk profile and hygiene concept

A protective factor that might have reduced the SARS-CoV-2 infection risk profile in our T_1_ cohort from the outset was the (for that time) high vaccination rate. Given that teachers are a common source of infection in the school setting, immunization of this cohort was highly relevant (30–32). Furthermore, studies have shown that adolescents have a transmission risk comparable to that of adults (30). Considering that the mean age of students was 14 years, a high vaccination rate in this age group can also be interpreted as a relevant protection factor. In addition, the high rates of mask usage before and during the trip, as well as reduced traveling during the Christmas holidays before the start of our study, may have decreased the initial SARS-CoV-2 risk profile. The isolation from locals and other visitors in the hostel was part of the T_1_ hygiene protocol and presumably served as an additional protective factor. However, compared to the previous summer trip (6), the higher occurrence of contact with individuals who tested positive for SARS-CoV-2 contributed to an increase in the risk profile for our January T_1_ cohort. As part of the hygiene protocol, the participants were urged to stay away from crowds between PCR testing and their departure, which was scheduled for 1 day later. Encouraging students and teachers to limit their contact during the 3–5 days before PCR testing, in a manner similar to a “light quarantine,” could enhance the reliability of the PCR pool test and reduce the risk of a positive finding during the trip. Furthermore, playing music outside to reduce the transmission risk when using wind instruments was not possible due to the weather conditions in winter. Nevertheless, the rules concerning regular intermittent ventilation, social distancing, and other preventive measures proved effective during the T_1_ trip as our positive-tested B_T1_ student, who played the trumpet, did not infect his co-musicians.

Overall, it must be kept in mind that a consequent testing regime can be considered a very effective tool for the successful implementation of a school music trip through the early identification of positive cases and thus breaking the possible infection chains. The question of whether a hygiene protocol has to be adopted, on top, can then be decided depending on the school administration’s views and the feasibility in post-pandemic times. When performing initial PoC-PCR tests, especially a mask requirement at the test site, for the short time before the results of the participants have arrived, would be conceivable to limit possible transmissions happening directly before the journey starts.

### 4.4 Impact on participants’ health

The analysis of reported physical symptoms amongst the children who participated in T_1_ did not show a unilateral pattern but rather varying developments during the study. Cold symptoms were reported significantly less often after attendance. This may be a consequence of the participants strictly adhering to the mandatory hygiene protocol. In contrast, a slight increase in specific symptoms, such as headache and cough, within the children’s group does not necessarily indicate increasing respiratory infections but can rather be interpreted as isolated symptoms from other causes. It is important to note that a higher incidence of respiratory tract symptoms is not uncommon among musicians who play wind instruments (33), which applies to 62 T_1_ participants in our study.

Findings regarding the effects of the T_1_ school music trip on the mental health of traveling children and teachers showed a slight rise in the GAD-7 anxiety score and an increased fear of contracting a SARS-CoV-2 infection. In addition, there was a significant increase in the category “perceived risk of infection” after the journey. This contrasts with the results of the summer cohort studied by Pudasaini et al., which showed a reduction in children’s anxiety levels (6). Although no specific reasons for the increase in fear and anxiety were elicited in our survey, it is possible that this dynamic may have occurred due to student B_T1_, who tested COVID-positive on the fourth day of the journey. Until then, he had been in contact with the majority of the travel group, although he was almost always wearing a mask. An increase in fear and anxiety might have directly resulted from this occurrence, suggesting that an implementation in summer months, when SARS-CoV-2 infections are less frequent, could be even more beneficial for mental health. In the first summer cohort, where no infections were detected during the journey, the symptoms of fear decreased significantly (6), supporting our hypothesis. However, the scores evaluating symptoms of depression and social isolation would have also been necessary to capture a more extensive view of the psychological status after the trip, as previous studies have shown a strong association between anxiety, depression, and social isolation in children during the pandemic (34). However, regardless of the occurrence of infections and the subsequent changes in fear and anxiety levels, it can be assumed that such music trips are perceived as an overall positive experience. This assumption may be drawn from the similar feedback that was given by both summer (6) and T_1_ winter cohorts, who expressed a desire for further participation in the future.

Overall, the observed changes in physical and psychological symptoms were primarily seen among students, while the teaching staff did not show similar alterations after participating in the trip. This could be explained by the more confined living conditions of the children during the T_1_ trip, as they shared one room with up to two other children, in contrast to the teachers who had their own chambers. Moreover, the teaching personnel was primarily responsible for didactic-organizational tasks and did not engage in playing (wind) instruments. These aspects may have made it easier to consistently wear masks and reduce possible category-one contacts. As a result, the teachers may have experienced lower anxiety levels and a reduced fear of infection compared to the student cohort.

## 5 Conclusion

Our study indicates that safe school music trips without SARS-CoV-2 transmission are possible by initially testing the whole traveling cohort using PCR pool tests. Amongst the two testing strategies that we used, PoC-PCR pool tests are considered the most time-efficient and location-flexible option, and this testing strategy could sustainably support a structured implementation of school leisure activities both during and after the pandemic. The individual cohort’s SARS-CoV-2 risk profile and possible adaptation of hygiene rules may also enhance safety. Further research on how to offer SARS-CoV-2-free recreational school activities for children and adolescents is needed to support programs and interventions on mental health prevention in children.

## Data Availability

The raw data supporting the conclusions of this article will be made available by the authors, without undue reservation.
