# Supplementary material for: SARS-CoV-2 testing strategies for a safe (post-)pandemic implementation of school music trips and their impact on participants’ health
Source: Front Public Health. 2025 Apr 8;13:1422243. doi: 10.3389/fpubh.2025.1422243 (PMC12011820; doi:10.3389/fpubh.2025.1422243)
Supplement: Supplementary file 1 [file Table_1.docx]

**Supplementary file 1: Hygiene concept**

**Mandatory:** during school music trip T_1_ from 04^th^ of January 2022 to 09^th^ of January 2022

**Recommended:** during school music trip T_2_ from 3^rd^ of January 2023 to 8^th^ of January 2023

Basic information:

80 students and 15 teachers are registered for the trip. Three full big bands are taking part. Most of the participants are fully vaccinated or have recovered from SARS-CoV-2. The destination is a large youth hostel in Saxony-Anhalt with over 250 beds.

Two different possible strategies were developed for the trip in advance in order to react quickly to relevant COVID-related developments such as a ban on accommodation. (In the following, only the version that was applied will be shown.)

**The present hygiene concept is coordinated with the model hygiene plan of the School Administration of the Berlin Senate, published on 10^th^ of November 2021.**

1. Structure:
   - Monday, 03^rd^ January: pool-polymerase chain reaction (PCR) tests
   - Discussion of bands at school, concrete tasks are given
   - Individual practice at home
   - Tuesday, 04^th^ January: departure via bus
   - Sunday, 09^th^ January: return journey via bus
   - Monday 10^th^ January till Friday 15^th^ January: daily antigen testing
2. Preparation for the trip, PCR testing for each person travelling with us:
   - In cooperation with the Charité – Universitätsmedizin Berlin, PCR tests are performed on Monday, 03^rd^ January. All students and teachers are obliged to undergo a testing procedure
   - If someone wants to attend the trip but does not participate in the corresponding study or they cannot be present in school that day, this person must privately organize a PCR test on Monday and is obliged to show the negative result to the team leader before departure
   - If a pool is positive, all members of this pool will be re-tested in the afternoon at the Charité – Universitätsmedizin Berlin to identify the infected person(s)
   - Only those who are tested negative via PCR, are allowed to attend the trip
   - Participants should stay away from crowds between testing and departure
   - All participants are informed in advance about the planned hygiene measures
3. Bus transfer:
   - The bus transfer is carried out in compliance with all hygiene regulations of the specific bus company
   - The first row behind the driver is to be kept free to ensure a minimum distance of 1.5 meters, as the driver cannot wear a mask
   - Boarding and exiting should be done in an orderly fashion and without crowding. In double-decker buses, people sitting on the upper deck are to board first. People sitting on the lower deck are to exit first
   - Medical masks are to be worn during the journey
   - Unprotected contact with other passengers in parking lots needs to be avoided
   - Going to the toilet is possible, shopping and eating at rest stops is to be avoided. FFP2 protective masks are to be worn there
4. Accommodation and behaviour on site
   - Our group is to stay together as a cohort with no outside contacts
   - Unprotected contact with other guests of the youth hostel and with staff members of the hostel is to be avoided
   - Visits in town, shopping trips to supermarkets etc. are to be avoided
   - Exploring the natural surroundings of the youth hostel in pairs or small groups is explicitly wished for
   - Accommodation and supplies are provided in accordance with the hygiene plans of the Saxony-Anhalt Youth Hostel Association
   - In the rooms of the youth hostel, protective medical masks are to be worn, and the usual hygiene regulations are to be followed
   - Fixed room allocations are assigned and no room swaps are to be made
   - Meals are taken separately from other guests
   - All participants must test themselves every day during the rehearsal week under supervision with antigen tests
5. Behaviour in case of a positive test
   - In case of a positive antigen test result, this person will be isolated in a separate room and will undergo a PCR test
   - The room neighbours remain in their room as category-one contacts and will also undergo PCR tests
   - In cooperation with the local health authority, the tour leader will decide whether there are any other category-one contacts
   - Until the test results are reported, the persons concerned will remain in isolation
   - If the test results are negative, the quarantine regulations will be lifted (in case they are fully vaccinated)
   - In the case of a positive PCR test, the pupil is to be picked up by their parents and needs to isolate at home. Appropriate measures will be taken for caregivers
   - If more than two participants of the travel group receive positive PCR results, the trip will be cancelled early
6. Sample situation
   - Before and after testing, the students and teachers must adhere to hand sanitation standards
   - Instruments and materials are only used by individual students. They are supposed to bring them to the music trip individually. It is not allowed to pass them on to other students
   - If possible, medical masks are to be worn during indoor rehearsals
   - If possible, rehearsals should take place with open windows
   - Sufficient ventilation breaks have to be made
   - During the first days, rehearsals are held individually or in small groups in the so-called section rehearsals. There are enough rooms and instructors available for this
   - In the course of the week, the small groups are put together to form larger groups (register rehearsals). Later on the trip, tutti-rehearsals of the individual ensembles are planned
   - According to our understanding, wind instruments (with exception of the flute) hardly spread any aerosols in the room. Therefore, the usual orchestra seating arrangements are set up
   - For wind instruments with condensation, suitable measures for absorbing the condensation will be prepared and implemented. The absorbed condensate will be disposed of appropriately
7. After the tour

- The follow-up of all participants, regarding possible SARS-CoV-2 infections, will take place as part of routine testing at the school up to and including Friday after return (14^th^ January). Only if infections occur, a re-testing of all trip participants with pooled PCR tests will be carried out on Friday

**Summary**

The greatest possible safety of the fellow travellers is ensured, because only negatively tested persons come along on the trip; the group remains together as a cohort and antigen tests are conducted daily.
